# Premature ventricular contractions originating from the left ventricular septum: Results of Radiofrequency Catheter Ablation in twenty patients

**DOI:** 10.1186/1471-2261-11-27

**Published:** 2011-06-02

**Authors:** Li Jia, Li Yue-Chun, Ji Kang-Ting, Zhou Na-Dan, Lin Jia-Xuan, Zhang Wen-Wu, Yang Peng-Lin, Tang Ji-Fei, Lin Jia-Feng

**Affiliations:** 1 Department of Cardiology, Second Affiliated Hospital of Wenzhou Medical College, Wenzhou 325000, China

## Abstract

**Background:**

RFCA has been established as an effective and curative therapy for severely symptomatic PVC from the outflow tract in structurally normal hearts. However, it is unknown whether PVCs originating from the left ventricular septum, are effectively eliminated by RFCA. This study aimed to investigate electrophysiologic characteristics and effects of Radiofrequency catheter ablation (RFCA) for patients with symptomatic premature ventricular contraction (PVC) originating from the left ventricular septum without including fascicular PVCs.

**Methods:**

Characteristics of body surface electrocardiogram (ECG) and electrophysiologic recordings endocardiogram in a successful RFCA target were analyzed in 20 patients with symptomatic PVCs originating from the left ventricular septum. RFCA was performed using pace mapping and activation mapping.

**Results:**

The QRS morphology of PVCs originating from the left ventricular septum is similar to that seen in fascicular tachycardia. Most of the PVCs originated from the left septum appears in the form of ventricular parasystole. The incidence of ventricular parasystole was 70%. Sustained ventricular tachycardia was not inducible by electrical stimulation and isoproterenol infusion in all 20 patients, ablation at the site recording the earliest Purkinje potential was not effective in all 20 patients, and Purkinje potentials were not identified at successful sites during point mapping. Sixteen patients were successful with RFCA using pace mapping and activation mapping, 3 failed, and 1 recurrent.

**Conclusion:**

Although the ECG characteristics of the PVCs arising from the left ventricular septum are similar to that seen in fascicular tachycardia, the electrophysiologic characteristics are different between the two types of PVCs. The distinguishing characteristic of the PVCs is that Purkinje potentials were not present at the site of successful ablation, suggesting a myocardial as opposed to fascicular substrate. RFCA is an effective curative therapy for symptomatic PVCs originating from the left ventricular septum (not from the left anterior and posterior fascicle).

## Background

Isolated premature ventricular complexes (PVCs) are the most common arrhythmias observed in patients without structural heart disease [1]. In recent years, radiofrequency catheter ablation (RFCA) has proven to be a safe and successful therapy for the arrhythmias [2-5]. PVCs mainly originate from the right ventricular outflow tract, with a small part of them originating from the left ventricular outflow tract, mitral and tricuspid valve annulus [6-11]. However, idiopathic PVCs that originated from the left ventricular septum, not involving the left anterior or posterior fascicle, have also been observed. The purpose of this study was to analyze electrophysiological characteristics and the outcome of catheter ablation for such PVCs originating from the left ventricular septum myocardium, without involving the Purkinje-fascicular fibers.

## Methods

### Study population

From September 2006 to May 2010, a total of 318 patients without structural heart disease were presented for catheter ablation for PVCs in our hospital. Only patients with idopthic PVCs from the left ventricular septum were enrolled in the present study. All patients were verified as having no structural heart disease, including coronary artery disease, valvular heart disease, congenital heart disease, left ventricle hypertrophy, and right ventricle abnormalities by routine biochemistry tests, X-ray, color echocardiography examination, exercise electrocardiogram testing, and/or cardiac catheterization with coronary angiography. Before RFCA, a 12-lead ECG was obtained, and 24 hours ambulatory ECG monitoring (Holter) was carried out at least once. The ECG was monitored for 24 hours just before catheter ablation.

### Inclusion criteria

The selection criteria of patients were the following reasons: (1) frequent PVCs occurrence, the average PVC count ≥ 10000 times/24 h; (2) inability of the patient to tolerate PVCs or unsuccessful treatment with at least two antiarrhythmic drugs; (3) no structural heart disease; and (4) consent for the catheter ablation procedure.

### ECG measurements

Twelve-lead electrocardiograms of the clinical arrhythmia were available for all patients with PVCs originating from the left ventricular septum. The analysis of ECG pattern was focusing on the following characteristics: (1) The QRS morphology of the PVC in all 12 leads, (2) the duration of QRS complex, (3) the axis deviation.

### Electrophysiologic study and RFCA

Electrophysiologic study was performed after withdrawal of all anti-arrhythmic drugs for at least five half-lives. Under fluoroscopic guidance, two quadripolar electrode catheter (Cordis, USA) were advanced from the right femoral vein and positioned in the right ventricular apex and near the His bundle. One additional catheter was inserted via the internal jugular vein and positioned in the coronary sinus. A 12-lead surface electrocardiogram was monitored and recorded on a multichannel oscilloscopic recorder. Programmed electrical stimulation was performed from right ventricular apex at basic drive cycle lengths of 600, 500, and 430 msec, delivering a maximum of three extrastimuli. A 7F quadripolar deflectable catheter was advanced into the left ventricle using retrograde aortic approach via the right femoral artery for mapping and ablation (Cordis, USA). The mapping/ablation catheter has a 4 mm distal electrode, with interelectrode spacing of 2-5-2 mm. Pace mapping and endocardial activation mapping were performed. If the clinical arrhythmia did not occur spontaneously and was not induced in the baseline, intravenous isoproterenol (2-4 μg/min) was administered to induce arrhythmia.

The target site of RFCA was determined by two steps:1) a careful search was firstly performed for Purkinje potentials preceding the onset of QRS during sinus rhythm along the left ventricular septum in all patients, and radiofrequency energy was delivered at sites demonstrating local endocardial recording with earliest presystolic Purkinje potentials during PVCs, or in sinus rhythm if there was insufficient ectopy to permit activation mapping; 2) next step is to obtain the complete or near complete pace mapping with the earliest local activation time if RFCA is not successful, and when the PVC occurred rarely during the electrophysiologic study, only pace mapping was performed, aiming to identify the site where pacing reproduced QRS morphology that is similar to the clinical PVC (≧ 11/12-lead concordance of major and minor deflections). After the target site was located, the energy of RFCA was delivered using maximum power of 40 W, maximum temperature of 60°C and impedance of 80-140 Ωin the temperature-controlled mode. If the PVC was terminated within 10 seconds or frequently PVCs and/or nonsustained ventricular tachycardia occurred during ablation at the target site, additional current was applied for another 60 to 180 seconds. Successful ablation was defined as complete elimination of spontaneous or inducible ventricular arrhythmias. Programmed electrical stimulation was repeated at 30 minutes after the last application of radiofrequency energy to confirm the absence of inducible ventricular arrhythmias before removing all catheters and sheaths. If PVC did not terminate within 10 s, the radiofrequency energy application was terminated and another target site was sought.

### Definition of successful ablation

Successful ablation was defined as the absence of spontaneous or induced clinical PVC and ventricular tachycardia, both with or without isoproterenol at the end of the procedure. Absence of PVC or ventricular tachycardia in ECG monitoring over 48 hours without anti-arrhythmia drugs. No recurrence of arrhythmia in the absence of anti-arrhythmic drug therapy during follow-up.

### Follow-Up

After RFCA, all patients underwent 72-hour ECG monitoring. Holter was carried out one week after RFCA. Patients were not given any antiarrhythmic drugs after RFCA, and underwent color echocardiography and Holter examination three month after RFCA. ECG echocardiography and 24-hour ECG monitoring were performed whenever the patient had symptoms suggestive of recurrence of ventricular arrhythmias..

### Definition of PVC originating from the left ventricular septum

PVC was considered to originate from the left ventricular septum, not from the left anterior and posterior fascicles, based on (1) the local endocardial recordings; (2) the characteristic of left ventricular septum location and motion (when viewed in the right and left anterior oblique fluoroscopic views after successful RFCA); (3) programmed electrical stimulation could not induce sustained ventricular tachycardia; (4) unsuccessful ablation at Purkinje potential site, and (5) purkinje potentials were not identified at successful sites during point mapping.

### Ethics Approval

Ethical approval was obtained from the Ethics Committee of the Second Affiliated Hospital of Wenzhou Medical College, and all patients gave informed consent before participation in the study.

## Results

### Study population

A total of 20 patients (10 males and 10 females) were enrolled in the present study. Among patients with idiopathic ventricular ectopy, the incidence of left septal ventricular arrhythmias was 6.29% (Table 1). PVCs in 8 of 318 patients originated from fascicles (Table 1). The mean PVC burden during the preoperative 24 hours ambulatory Holter monitoring was 17774.05 ± 5861.66.

**Table 1:** PVC origin and results of RFCA for idiopathic ventricular arrhythmias

Arrhythmia origin	No.(%)	Success (%)
**LV septum**	20 (6.29)	16 (80.00)

**Anterosuperior septum**	11 (3.46)	9 (81.82)

**Posteroinferior septum**	9 (2.83)	7 (77.78)

**Fascicle**	8 (2.52)	8 (100)

**RVOT**	215 (67.61)	204 (94.88)

**PA**	11 (3.46)	11 (100.00)

**Tricuspid annulus**	26 (8.18)	23 (88.46)

**Aortic sinus of Valsalva**	20 (6.29)	15 (75.00)

**LVOT**	4 (1.26)	4 (100.00)

**Mitral annulus**	4(1.26)	4(100.00)

**LV epicardium**	5(1.57)	3 (60.00)

**Others (RVIT 3,LV Free wall 3)**	5 (1.57)	4 (80.00)

**Total**	318 (100.00)	351 (91.82)

RVOT or LVOT, the right or left ventricular outflow tract, respectively; PA, pulmonary artery; PVCs, premature ventricular complexes; LV, left ventricular; RVIT, right ventricular inflow tract; LV epicardium, Idiopathic ventricular arrhythmias that could not be ablated with RFCA from the left sinus of Valsalva despite earliest ventricular activation being recorded in the left sinus of Valsalva or that could be ablated within coronary venous system were classified as originating from the LV epicardium in the present study.

#### 1) Electrophysiologic findings

PVCs in 20 patients originated from left septum, including 11 from anterosuperior septum, 9 from posteroinferior septum. Table 2 lists the baseline characteristics. The PVC occurred spontaneously in 18 patients and was induced by bolus injection of isoproterenol (2 μg) in two patients during the electrophysiologic study. Sustained ventricular tachycardia was not inducible by electrical stimulation and isoproterenol infusion in any patient.

**Table 2:** Baseline patients characteristics

Patient	Age (years)	Sex	PVC count (number/24 h)	Number of AADs used	Comorbidities	LVEF (%)	Ventricular parasystolic activity	Origin of PVC (septum)	RF Lesions prior to success
1	14	M	23764	3	none	65	yes	posteroinferior	5

2	54	M	24316	2	none	62	yes	anterosuperior	3

3	72	M	16243	2	none	62	no	posteroinferior	6

4	47	F	24318	2	none	68	yes	anterosuperior	2

5	65	M	11236	2	none	64	yes	posteroinferior	3

6	14	M	10243	3	none	72	no	anterosuperior	5

7	76	M	16452	2	none	60	yes	posteroinferior	5

8	27	F	26742	3	none	70	no	anterosuperior	6

9	24	M	18631	3	none	67	yes	anterosuperior	4

10	37	F	19632	2	none	66	yes	anterosuperior	3

11	17	F	12763	2	none	66	no	posteroinferior	4

12	39	M	16537	2	none	63	yes	posteroinferior	5

13	45	F	12344	2	none	58	yes	posteroinferior	3

14	38	M	15891	2	none	61	yes	anterosuperior	2

15	48	F	31463	2	none	63	yes	anterosuperior	2

16	69	F	15409	2	none	57	yes	anterosuperior	3

17	30	F	10858	2	none	68	no	anterosuperior	4

18	28	F	11695	2	none	71	yes	posteroinferior	6

19	27	M	16932	2	none	66	yes	posteroinferior	3

20	35	F	20012	2	none	69	no	anterosuperior	2

AADs = antiarrhythmic drugs, LVEF = left ventricular ejection fraction

#### 2) Effect of RFCA

**Figure 1:**
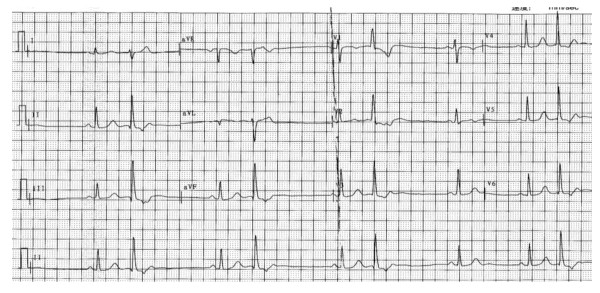
**Endocardial recordings of successful ablation target site originating from left anterosuperior septum**. The left panel shows that the local ventricular activation time recorded at the ablation site that preceded the onset of the QRS complex was 33 ms and Purkinje potentials were not present at the site of successful ablation. The middle panel shows that pace map at the ablation site provides an identical (12/12) match with the clinical PVC morphology. The right panel shows double potential in front of PVC or VT during ablation ABL, ablation catheter.

**Figure 2:**
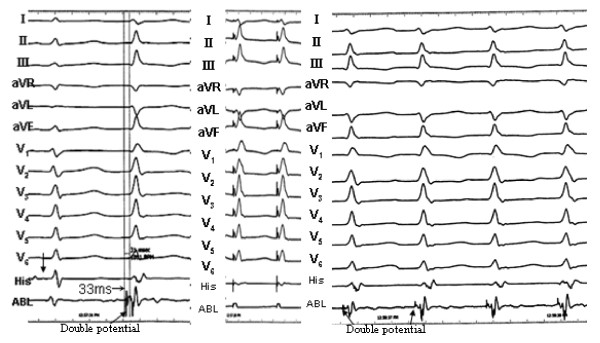
**Termination of the PVCs originating from left anterosuperior septum during RF application at the site**. The ventricular tachycardia appeared at the beginning of ablation. The middle panel shows the fluoroscopic position of the ablation site. RAO, right anterior oblique projection; LAO, left anterior oblique projection.

**Figure 3:**
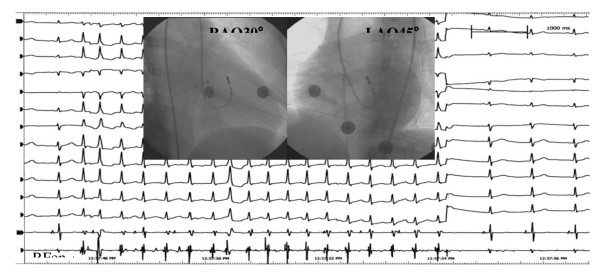
**The middle panel show endocardiogram of successful ablation target site originating from left posteroinferior septum**. The left panels show ECG during ventricular pacing. The right panels show that the local ventricular activation time recorded at the successful ablation target site that preceded the onset of the QRS complex was 38 ms.

Ablation at the site recording the earliest Purkinje potential was not effective in all 20 patients. By using pace mapping technique and/or activation mapping, RFCA was applied in 20 patients, 17 treatments of which were successful (immediate ablation success rate 85%, Figure 1, Figure 2, Figure 3). Purkinje potentials were not identified at successful sites during point mapping. The local ventricular activation time recorded at successful ablation target sites that preceded the onset of the QRS complex was (31.78 ± 3.27) ms. All pace mappings were perfect (≧11/12). Operations came off smoothly with no ablation related complications. Patients have been followed-up for a mean follow-up period of 20.2 months without antiarrhythmic medications. Only one patient had recurrent ventricular arrhythmia at 16 hours after RFCA. The results of RFCA are summarized in Table 3.

**Table 3:** The results of RFCA

	Anterosuperior group (n = 11)	Posteroinferior group (n = 9)	*P value
Procedure duration (min)	72.29 ± 9.71	80.57 ± 18.32	>0.05
Radiation exposure time (min)	13.53 ± 5.88	14.29 ± 6.95	>0.05
Time of the earliest ventricular activation preceding the QRS onset (ms)	30.80 ± 3.70	33.20 ± 2.95	>0.05
RF lesions prior to success	3.3 ± 1.3	4.3 ± 1.4	>0.05
Immediate ablation success rate	81.8%	88.9%	>0.05
Recurrent rate (%)	0/9 (0%)	1/8 (12.5%)	>0.05
Follow-up time (month)	19.43 ± 15.68	21.14 ± 16.21	>0.05
The average cost of ablation per patient (RMB)	15740.00 ± 2219.16	16440.00 ± 2619.16	>0.05

* P values were compared between the two groups. RMB: Ren Min Bi or China Yuan (China's Currency).

#### 3) 12-lead ECG characteristics of PVC originating from the left ventricular septum

**Figure 4:**
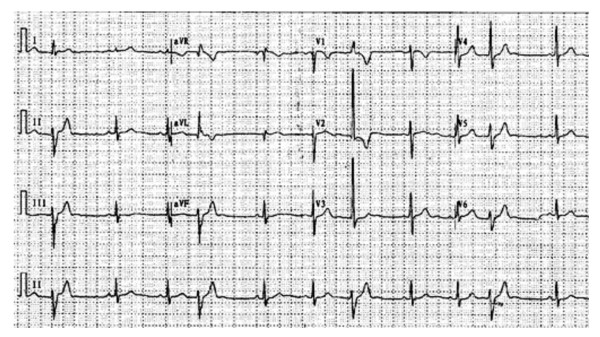
**Representative 12-lead ECG characteristics of ventricular arrhythmia originating from left anterosuperior septum**.

**Figure 5:**
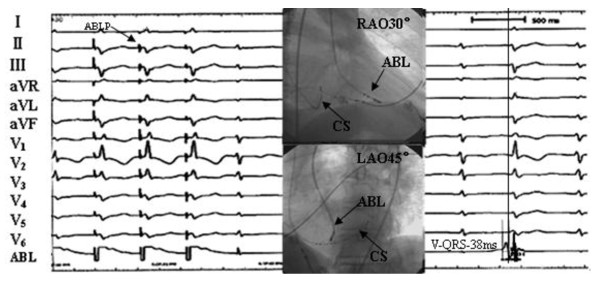
**The representative 12-lead ECG characteristics of ventricular arrhythmia originating from left posteroinferior septum**.

The electrocardiographic patterns of PVCs are different at the different sites of origin of left ventricular septum (Table 4). For PVCs originating from left anterosuperior septum, their QRS complex morphology were qR or qRs in leads II, III, aVF, Rs (R/s > 1) in leads V5~V6, rs(S) in leadsI, aVL and Qr in lead aVR (Figure 4); For PVCs originating from left posteroinferior septum, their QRS complex morphology were rS in leadsII, III, aVF, R(r)S(R/S < 1) in leads V5~V6, qR(s) in leadsI,aVL and qR in lead aVR (Figure 5). In 14 of the 20 subjects, the PVCs originating from the left ventricular septum appear in the form of the ventricular parasystolic rhythm (Table 2). The incidence rate of ventricular parasystole was 70% (14/20).

**Table 4:** The ECG characteristics of PVCs originating from different sites of origin of left ventricular septum

Group	I,aVL	II, III,aVF	aVR	V1	V2-V4	V5-V6	axis	QRS duration
anterosuperior	rs or rS	qR or qRs	Qr	rSR or R	qR/R/RS	R or Rs	Right	99.8 ± 8.7 ms

posteroinferior	Rs or qR	rS	qR	R	Rs	RS	Left	116.7 ± 13.6 ms

## Discussion

The main findings of this study are that PVCs arising from the left ventricular septum can be safely and effectively eliminated with RFCA, the localization of optimal ablation site of the PVCs is different with the typical location for ectopy originating from the classical fascicular sites. The distinguishing characteristic of this arrhythmia is that Purkinje potentials were not present at the site of successful ablation, suggesting a myocardial as opposed to fascicular substrate. This is the first report that demonstrated that RFCA eliminates PVCs originating from the left ventricular septum (not from classical fascicular sites) by pace mapping and activation mapping, not by searching for Purkinje potential in the left ventricular septum, and found that most cases with the PVCs arising from the left ventricular septum behaved with parasystolic activity.

In this study, we found that PVCs originating from the left ventricular septum have distinctive ECG characteristics. First, the electrocardiographic patterns are different at the different sites of origin of left septal PVC. For PVCs originating from left anterosuperior septum, their QRS complex morphology were qR or qRs in leads II, III, aVF, Rs (R/s > 1) in leads V5~V6, rs(S) in leadsI, aVL and Qr in lead aVR. For PVCs originating from left posteroinferior septum, their QRS complex morphology were rS in leads II, III, aVF, R(r)S (R/S < 1) in leads V5~V6, qR(s) in leadsI,aVL and qR in lead aVR. The QRS morphology of PVCs originating from the left ventricular septum is similar to that seen in fascicular tachycardia [12]. Why are there the similarity of the morphology of the premature septal beats and fascicular beats? We suggest that the PVCs may originate from the septal myocardium near by the Purkinje network. Second, most of the PVCs originated from the left septum appear in the form of ventricular parasystole, which is different with fascicular PVCs. In the study, the incidence of ventricular parasystole was 70%. This suggests that the mechanism of the two types of PVCs may be different.

In this study, ventricular parasystole was often observed, sustained ventricular tachycardia was not inducible by electrical stimulation and isoproterenol infusion in all 20 patients, ablation at the site recording the earliest Purkinje potential was not effective in all 20 patients, and Purkinje potentials were not identified at successful sites during point mapping. These suggest enhanced antomaticity, but not reentry as the most likely mechanism of PVCs originating from the left ventricular septum in the study [13,14]. The electrophysiologic characteristics of the PVCs originating from the left ventricular septum in the study were different with those from the classical fascicular sites. Therefore, the form of ectopy may occur from the myocardium of the septum, instead of the Purkinje network.

In this study, we demonstrated PVCs arising from the left ventricular anterosuperior and posteroinferior septum can be safely and effectively eliminated with catheter ablation techniques. The immediate ablation success rate was 85%, and the chronic success rate was 80% (16 of 20 patients) during a mean follow-up period of 20.2 months. One patients experienced significant recurrence of PVCs with associated symptoms. The overall success rate for ablation of PVCs originating from the left ventricular septum in the present study nearly corresponded with the results of previous reports for ablation of PVCs originating from RVOT and/or other sites of origin [4,15,16]. The mapping techniques in our study were basically the same as those described in previous studies [4,5], which included pace mapping and activation mapping. No significant complications were observed in our patient group confirming the safety of the procedure.

### Study limitations

First, the mechanism of the PVCs arising from the left ventricular septum is thought to be due to enhanced antomaticity, but not reentry, but this remains speculative in the limited clinical study. Second, although the PVCs may occur from the myocardium of the septum, instead of the Purkinje network, it is unknown that how distributed were these septal sites. To increase the accuracy of our study, our results need to be confirmed in a larger prospective randomized patient population.

## Conclusions

Although the ECG characteristics of the PVCs arising from the left ventricular septum are similar to that seen in fascicular tachycardia, the electrophysiologic characteristics are different between the two types of PVCs. Most cases with the PVCs arising from the left ventricular septum behaved with parasystolic activity. The results of the present study confirm the high success rate and safety of RFCA using conventional techniques in the management of PVCs originating from the left ventricular septum.

## Competing interests

The authors declare that they have no competing interests.

## Authors' contributions

LJF and LYC designed the whole study, LJF, LJ, LYC, JKT, ZND, LJX, ZWW, YPL, and TJF performed the experiment, LJF and LYC wrote the paper. All authors read and approved the final manuscript.

## Pre-publication history

The pre-publication history for this paper can be accessed here:

http://www.biomedcentral.com/1471-2261/11/27/prepub
